# Endoscopic Retrograde Cholangiopancreatography for Choledocholithiasis: Clinical Outcomes and Predictors of Complications

**DOI:** 10.7759/cureus.95865

**Published:** 2025-10-31

**Authors:** Muhammad Khizar Badar, Jasim Ali, Baseera Imran, Muhammad Usama Rasheed, Muhammad Umar Kaleem, Ali Raza

**Affiliations:** 1 Internal Medicine, Imran Idrees Teaching Hospital, Sialkot, PAK; 2 Internal Medicine, Aziz Bhatti Shaheed Teaching Hospital, Gujrat, PAK; 3 Physiology, Ameer-ud-Din Medical College, Lahore, PAK; 4 Internal Medicine, Farooq Hospital, Lahore, PAK; 5 Internal Medicine, Akhtar Saeed Medical and Dental College, Lahore, PAK; 6 Emergency Medicine, Shalamar Hospital, Lahore, PAK

**Keywords:** common bile duct, endoscopic retrograde cholangiopancreatography, lithotripsy, pancreatitis, stenting

## Abstract

Background

Common bile duct (CBD) stones are a frequent cause of biliary obstruction and may lead to life-threatening complications such as cholangitis, pancreatitis, and obstructive jaundice if not promptly managed. Endoscopic retrograde cholangiopancreatography (ERCP) has largely replaced open and laparoscopic bile duct exploration as the preferred therapeutic approach due to its minimally invasive nature, shorter hospital stay, and high efficacy. However, its success and safety depend on multiple clinical and procedural factors that require evaluation in local settings.

Objective

This study aims to evaluate the outcomes of ERCP for CBD stones in terms of efficacy, complications, and recurrence and to identify factors associated with post-ERCP adverse events.

Methods

This retrospective observational study was conducted at Akhtar Saeed Trust Hospital, Lahore, Pakistan, from January 2022 to January 2025, including 129 patients who underwent ERCP for suspected or confirmed CBD stones. Demographic, clinical, and procedural data were analyzed. Descriptive statistics were calculated for continuous variables (mean ± SD) and categorical variables (n, %). Comparative analyses between patients with and without complications were performed using an independent-sample t-test, Chi-square, or Fisher’s exact test, and binary logistic regression was applied to determine predictors of complications. A p-value < 0.05 was considered statistically significant.

Results

The mean age of patients was 52.6 ± 13.4 years, with 76 (58.9%) females. The most common presenting symptom was abdominal pain, 91 (70.5%), followed by jaundice, 66 (51.2%). Complete stone clearance was achieved in 116 patients (89.9%) during the first ERCP session. Post-ERCP complications occurred in 17 patients (13.2%), most commonly pancreatitis in nine patients (7.0%), bleeding in four (3.1%), cholangitis in three (2.3%), and perforation in one patient (0.8%). Multiple stones were significantly associated with complications (OR = 2.89, 95% CI = 1.05-7.98, p = 0.041), and a hospital stay of more than five days was strongly predictive of adverse outcomes (OR = 6.12, 95% CI = 1.98-18.9, p = 0.002). No procedure-related mortality occurred, and recurrence of CBD stones was observed in seven (5.4%) cases during follow-up.

Conclusion

ERCP is a highly effective and safe therapeutic modality for CBD stones, providing high clearance rates with acceptable complications and no mortality. The presence of multiple stones and prolonged hospitalization were associated with increased risk of adverse events. Outcomes were influenced by stone size, number, and complexity, highlighting the importance of individualized patient assessment and careful procedural planning. ERCP continues to serve as the cornerstone of choledocholithiasis management, offering shorter hospital stays, faster recovery, and lower morbidity compared to surgical alternatives.

## Introduction

Common bile duct (CBD) stones, also referred to as choledocholithiasis, represent a prevalent clinical problem in hepatobiliary medicine. They often occur as sequelae to gallstones that migrate into the bile duct but may also form de novo within the biliary tree [1]. Epidemiological research indicates that CBD stones occur in approximately 10%-15% of all patients with gallstones, demonstrating a significant overlap between cholelithiasis and choledocholithiasis [2]. Clinically, patients may remain asymptomatic or present with acute complications such as cholangitis, biliary pancreatitis, or obstructive jaundice. Early diagnosis and appropriate intervention are essential to reduce morbidity and mortality associated with these conditions.

Historically, the treatment of choice was open choledocholithotomy, later replaced by laparoscopic common bile duct exploration (LCBDE) [3]. Although effective, these surgical approaches were associated with increased risk of bile duct injury, longer hospital stays, and delayed recovery [4]. The introduction of endoscopic retrograde cholangiopancreatography (ERCP) in the 1960s revolutionized management, providing a minimally invasive alternative that combined both diagnostic and therapeutic capabilities [5]. When performed with endoscopic sphincterotomy (EST), balloon or basket extraction, and mechanical lithotripsy, ERCP achieves >90% success rates in ductal clearance [6,7]. It offers shorter recovery time, less postoperative pain, and lower morbidity, especially in elderly or comorbid patients who are poor surgical candidates [8].

Despite these advantages, ERCP is not without risks. The most common and feared complication is post-ERCP pancreatitis (PEP), reported in 3%-10% of cases [9]. Other complications include bleeding, duodenal or bile duct perforation, cholangitis, and cardiopulmonary events related to sedation. Although ERCP-related mortality is typically below 1%, the associated morbidity underscores the importance of meticulous patient selection and technical expertise. Regional data from South Asia, including Pakistan, are limited, but available reports suggest comparable complication rates to those seen internationally, emphasizing the need for local studies to identify population-specific risk factors.

In recent years, attention has focused on preventive strategies to mitigate ERCP-related complications. The use of rectal nonsteroidal anti-inflammatory drugs (NSAIDs) such as indomethacin or diclofenac, pancreatic duct stenting in high-risk patients, and pre-procedure risk stratification have demonstrated efficacy in reducing PEP incidence. Incorporating these strategies into local practice requires robust evidence derived from regional cohorts.

Long-term outcomes after ERCP also merit consideration. Stone recurrence rates range from 4% to 24%, influenced by factors such as biliary stasis, sphincter of Oddi dysfunction, anatomical variations, and periampullary diverticula [10]. Recurrent interventions may be required for difficult or recurrent stones, raising questions about the need for structured surveillance and prophylactic measures.

Alternative modalities such as LCBDE, endoscopic ultrasound (EUS)-guided biliary drainage, and peroral cholangioscopy have expanded the therapeutic spectrum for CBD stones [11,12]. While these offer distinct advantages such as simultaneous management of gallbladder stones in LCBDE or improved visualization with cholangioscopy, their application is often limited in developing countries due to cost, equipment availability, and operator expertise. Consequently, ERCP remains the most accessible and widely practiced intervention, especially in tertiary care settings across Pakistan and similar regions.

Given the limited local data and the continuing importance of ERCP in managing biliary disorders, this study aims to evaluate the frequency, types, and predictors of post-ERCP complications in a tertiary care hospital. The findings are expected to provide region-specific insights that could guide risk stratification, inform preventive measures, and contribute to improving procedural safety and patient outcomes.

Objective

This study aims to evaluate the outcomes of ERCP for CBD stones in terms of efficacy, complications, and recurrence and to identify factors associated with post-ERCP adverse events.

## Materials and methods

Methodology

This was a retrospective observational study conducted at Akhtar Saeed Trust Hospital, Lahore, Pakistan, from January 2022 to January 2025. The study protocol was reviewed and approved by the Institutional Review Board (IRB) of Akhtar Saeed Medical and Dental College. As this was a retrospective chart review, the requirement for individual informed consent was waived by the ethics committee. Patient data were anonymized to ensure confidentiality and compliance with the Declaration of Helsinki (2013 revision).

A total of 129 patients were included. The required sample size, calculated using the WHO calculator, was 115 patients (assuming a clearance rate of 90%, 95% CI, ±5.5% precision) [13]. Our final cohort of 129 exceeded this threshold, providing approximately 5%-6% precision for both the clearance and complication rates, which is considered sufficient for an observational outcomes study.

Inclusion and exclusion criteria

Patients aged 18 years and above who underwent ERCP for suspected or confirmed CBD stones and had complete medical records with available follow-up data were included in the study. Patients were excluded if ERCP was performed for indications other than CBD stones, such as malignant biliary obstruction or benign biliary strictures, or if they had incomplete records or were lost to follow-up.

Diagnosis of CBD Stones

Diagnosis was established using abdominal ultrasound, magnetic resonance cholangiopancreatography (MRCP), or direct fluoroscopic evidence during ERCP of one or more filling defects consistent with calculi.

Definitions of Key Outcomes

Stone clearance: Complete absence of stones on post-procedural cholangiogram with free contrast flow into the duodenum.

Complications: Defined according to Cotton et al.'s classification of ERCP-related adverse events, including PEP, bleeding, perforation, and cholangitis [14].

PEP: New or worsened abdominal pain with serum amylase ≥3 times the upper limit of normal within 24 hours post-procedure, requiring ≥2 days of hospitalization.

Recurrence: Detection of CBD stones on follow-up imaging or ERCP at least three months after initial clearance.

Data collection

A non-probability consecutive sampling technique was used to include all eligible patients who underwent ERCP for suspected or confirmed choledocholithiasis during the study period. Medical records and endoscopy reports were reviewed to collect information on demographic details (age and gender), clinical presentation (jaundice, abdominal pain, cholangitis, and pancreatitis), laboratory parameters, radiological findings, procedural details, and therapeutic interventions performed. Procedural outcomes included stone clearance rates, requirement of additional interventions (mechanical lithotripsy and stenting), and need for repeat ERCP. Post-procedural complications such as pancreatitis, bleeding, perforation, and cholangitis were also recorded.

Data analysis

Data were entered and analyzed using Statistical Package for the Social Sciences (SPSS) version 26.0 (IBM Corp., Armonk, NY). Descriptive statistics were calculated for demographic and clinical variables. Continuous variables were presented as mean ± standard deviation (SD), while categorical variables were expressed as frequencies and percentages. Comparative analyses between patients with and without complications were performed using an independent-sample t-test for continuous variables, and Chi-square or Fisher’s exact test for categorical variables. Variables with p < 0.10 on univariate analysis were entered into a binary logistic regression model to identify independent predictors of post-ERCP complications. A p-value < 0.05 was considered statistically significant.

Missing data were handled using pairwise deletion, excluding only those variables with incomplete entries for specific analyses.

## Results

Data were collected from 129 patients undergoing ERCP for CBD stone extraction. The mean age of participants was 52.6 ± 13.4 years, with a slight predominance of females (58.9%) over males (41.1%). The most frequent presenting complaint was abdominal pain (70.5%), followed by jaundice (51.2%), cholangitis (20.9%), and acute pancreatitis (14.0%). Regarding stone characteristics, 55.8% of patients had stones smaller than 10 mm, while 44.2% had stones ≥ 10 mm. A single stone was identified in 62.8%, whereas multiple stones were observed in 37.2% of patients; impacted stones were seen in 11.6%.

Procedurally, complete ductal clearance was achieved during the first ERCP in 116 patients (89.9%), while 13 patients (10.1%) required additional interventions, including repeat ERCP (6.2%) or biliary stenting (3.9%) (Table 1).

**Table 1:** Baseline characteristics of patients (n = 129) Data are presented as mean ± standard deviation (SD) for continuous variables and n (%) for categorical variables. ERCP: Endoscopic retrograde cholangiopancreatography.

Variable	Value
Age (years), mean ± SD	52.6 ± 13.4
Gender	
– Male	53 (41.1%)
– Female	76 (58.9%)
Clinical presentation	
– Abdominal pain	91 (70.5%)
– Jaundice	66 (51.2%)
– Cholangitis	27 (20.9%)
– Acute pancreatitis	18 (14.0%)
Stone characteristics	
– Stone size < 10 mm	72 (55.8%)
– Stone size ≥ 10 mm	57 (44.2%)
– Single stone	81 (62.8%)
– Multiple stones (> 1)	48 (37.2%)
– Impacted stone	15 (11.6%)
ERCP procedural outcome	
– Complete clearance (first ERCP)	116 (89.9%)
– Incomplete clearance	13 (10.1%)
– Required repeat ERCP	8 (6.2%)
– Biliary stenting	5 (3.9%)

A total of 17 patients (13.2%) developed post-procedural complications, defined according to Cotton et al.'s classification. The most frequent adverse event was PEP, observed in nine patients (7.0%), followed by bleeding in four (3.1%), cholangitis in three (2.3%), and duodenal perforation in one patient (0.8%). No procedure-related mortality was reported. During follow-up, seven patients (5.4%) experienced recurrence of CBD stones, all successfully managed with repeat ERCP (Table 2).

**Table 2:** Post-ERCP complications and outcomes Complications and outcomes are presented as n (%). ERCP: Endoscopic retrograde cholangiopancreatography; CBD: Common bile duct.

Complication	n (%)
Post-ERCP pancreatitis	9 (7.0%)
Bleeding	4 (3.1%)
Cholangitis	3 (2.3%)
Perforation	1 (0.8%)
Total complications	17 (13.2%)
Recurrence of CBD stones	7 (5.4%)
Managed successfully with ERCP	7 (100%)

Endoscopic sphincterotomy was performed in 105 (81.4%) patients, balloon/basket extraction in 92 (71.3%), and mechanical lithotripsy in eight (6.2%). Biliary stenting was required in 5.4%, while nasobiliary drainage was placed in three patients (2.3%). The distribution of interventions between those with and without complications is summarized in Table 3. No statistically significant differences were observed among procedural techniques (Table 3).

**Table 3:** Interventions performed during ERCP Chi-square or Fisher’s exact test was applied. p < 0.05 is considered significant. ERCP: Endoscopic retrograde cholangiopancreatography.

Intervention	No complications (n = 112)	Complications (n = 17)	p-value
Endoscopic sphincterotomy	92 (82.1%)	13 (76.5%)	0.58
Balloon/basket extraction	80 (71.4%)	12 (70.6%)	0.94
Mechanical lithotripsy	6 (5.4%)	2 (11.8%)	0.27
Biliary stenting	3 (2.7%)	2 (11.8%)	0.09
Nasobiliary drainage	2 (1.8%)	1 (5.9%)	0.34

The mean hospital stay was significantly longer in patients with complications (5.1 ± 1.6 days) than in those without (3.3 ± 1.4 days; p < 0.001). Readmission within 30 days occurred in six patients with complications (35.3%) versus five (4.5%) without, also statistically significant (p < 0.001). No in-hospital or post-discharge mortality occurred (Table 4).

**Table 4:** Hospitalization and outcomes Independent samples t-test and Chi-square/Fisher’s exact test was applied. *p ≤ 0.05 is considered significant.

Parameter	No complications (n = 112)	Complications (n = 17)	p-value
Hospital stay (days), mean ± SD	3.3 ± 1.4	5.1 ± 1.6	<0.001*
Readmission within 30 days	5 (4.5%)	6 (35.3%)	<0.001*
Mortality	0 (0%)	0 (0%)	–

Univariate logistic regression identified multiple stones (OR = 2.89, 95% CI = 1.05-7.98; p = 0.041) and hospital stay > five days (OR = 6.12, 95% CI = 1.98-18.9; p = 0.002) as significant factors associated with complications. However, the latter was likely a reverse-causation effect, reflecting complication-related prolonged hospitalization (Table 5).

**Table 5:** Univariate logistic regression for predictors of post-ERCP complications Odds ratios (OR) with 95% confidence intervals (CI) were calculated using binary logistic regression analysis. *p ≤ 0.05 is considered significant. ERCP: Endoscopic retrograde cholangiopancreatography.

Variable	OR (95% CI)	p-value
Age ≥ 60 years	1.42 (0.51–3.96)	0.49
Female gender	0.79 (0.29–2.16)	0.65
Stone size ≥ 10 mm	1.98 (0.72–5.43)	0.18
Multiple stones (> 1)	2.89 (1.05–7.98)	0.041*
Impacted stone	2.76 (0.79–9.68)	0.11
Biliary stenting required	4.94 (0.77–31.4)	0.08
Hospital stay > 5 days	6.12 (1.98–18.9)	0.002*

On multivariate adjustment for confounders (age, gender, stone size, and impacted stones), multiple stones remained the only independent predictor of complications (adjusted OR = 2.76, 95% CI = 1.01-7.56; p = 0.047) (Table 6).

**Table 6:** Multivariate logistic regression analysis for predictors of post-ERCP complications Odds ratios (OR) with 95% confidence intervals (CI) were calculated using binary logistic regression analysis. *p ≤ 0.05 is considered significant. ERCP: Endoscopic retrograde cholangiopancreatography.

Variable	Adjusted OR (95% CI)	p-value
Age ≥ 60 years	1.29 (0.43–3.86)	0.65
Female gender	0.82 (0.28–2.38)	0.71
Stone size ≥ 10 mm	1.64 (0.57–4.67)	0.36
Multiple stones (> 1)	2.76 (1.01–7.56)	0.047*
Impacted stone	2.12 (0.62–7.25)	0.23

Over a median follow-up of nine months (IQR 6-14), seven patients (5.4%) experienced recurrence of CBD stones. Recurrence was significantly higher among patients with multiple stones (10.4% vs. 2.5%; p = 0.03) and those who underwent biliary stenting (42.9% vs. 4.9%; p = 0.04). No significant association was found for age, gender, or stone size (Table 7).

**Table 7:** Factors associated with recurrence of CBD stones Chi-square or Fisher’s exact test was used. *p ≤ 0.05 is considered significant. CBD: Common bile duct.

Variable	Recurrence (n = 7)	No recurrence (n = 122)	p-value
Age ≥ 60 years	3 (42.9%)	46 (37.7%)	0.78
Female gender	4 (57.1%)	72 (59.0%)	0.91
Stone size ≥ 10 mm	4 (57.1%)	53 (43.4%)	0.49
Multiple stones (> 1)	5 (71.4%)	43 (35.2%)	0.03*
Impacted stone	2 (28.6%)	13 (10.7%)	0.18
Biliary stenting performed	3 (42.9%)	6 (4.9%)	0.04*

## Discussion

This retrospective study evaluated the therapeutic outcomes of ERCP in 129 patients with CBD stones, focusing on efficacy, complications, and recurrence. The findings reaffirm that ERCP remains a highly effective and safe modality for biliary stone management. The first session's complete stone clearance rate was 89.9%, consistent with previously reported rates of 85%-95% in high-volume centers [15-17]. The slightly lower success rate observed in our cohort may be attributed to the presence of large, multiple, or impacted stones, which are established predictors of technical difficulty and procedural failure.

Regarding adverse events, the overall complication rate was 13.2%, with PEP being the most frequent (7.0%). This aligns with the international incidence range of 3%-10% reported in prior literature [15-17]. Other adverse events included bleeding (3.1%), cholangitis (2.3%), and duodenal perforation (0.8%), all within expected ranges. Importantly, there was no procedure-related mortality, supporting the safety of ERCP when performed by trained endoscopists under standardized protocols. These results underscore the importance of meticulous patient selection and adherence to preventive strategies, such as prophylactic rectal NSAID administration or pancreatic duct stenting in high-risk individuals, to reduce the incidence of PEP.

In our study, patients with multiple stones and those with a prolonged hospital stay (>five days) were significantly more likely to experience complications. These findings reflect the additive procedural complexity and prolonged biliary instrumentation associated with multiple calculi. Integrating these predictors into pre-procedural risk assessment may improve patient counseling and allow better resource allocation for high-risk cases.

The recurrence rate of CBD stones during follow-up was 5.4%, which is lower than the 4%-24% recurrence range reported in the literature [18,19]. This relatively low recurrence may reflect the shorter follow-up duration and effective complete duct clearance achieved during initial ERCP. Nevertheless, stone recurrence emphasizes the importance of long-term surveillance, particularly in patients with gallbladder in situ, who remain at risk for re-migration of stones. Timely cholecystectomy in such patients can prevent recurrent choledocholithiasis.

The mean hospital stay in this study was 3.6 ± 1.5 days, notably shorter than durations typically observed following surgical interventions. This supports the cost-effectiveness and patient-centered benefits of ERCP, including early recovery and reduced healthcare burden. The 30-day readmission rate of 8.5%, mainly due to recurrent symptoms or mild complications, was considerably lower than surgical readmission rates cited in comparative literature [20,21].

In comparing alternative modalities, LCBDE remains a valid one-stage surgical option, particularly when performed alongside cholecystectomy. However, it demands higher technical expertise, longer operative times, and costlier equipment. In contrast, ERCP is widely accessible, minimally invasive, and highly adaptable, making it the preferred intervention in most clinical contexts. EUS and cholangioscopy offer complementary diagnostic precision but are less feasible in resource-constrained environments. Therefore, optimizing ERCP through pre-procedural imaging, awareness of anatomical variations, and individualized patient assessment is essential for minimizing complications and maximizing therapeutic success [22].

This study has several limitations. First, its retrospective, single-center design introduces potential selection and information bias, limiting generalizability. Second, incomplete documentation of prophylactic measures (e.g., rectal NSAID use, pancreatic stent placement) may have influenced complication rates. Third, short and variable follow-up likely underestimated the true long-term recurrence of CBD stones. Fourth, operator experience and procedural nuances were not standardized or included in multivariable analyses, allowing possible confounding. Finally, the absence of a comparative (surgical or alternative endoscopic) arm limits causal inferences regarding predictors of complications.

Future prospective multicenter studies with standardized protocols, extended follow-up, and adjusted outcome modeling are warranted to clarify independent predictors of adverse outcomes and refine ERCP risk stratification.

## Conclusions

Endoscopic retrograde cholangiopancreatography (ERCP) is a highly effective and safe therapeutic option for managing CBD stones. In this tertiary-center cohort from Pakistan, ERCP achieved a high first-session clearance rate (89.9%) with an acceptable complication rate (13.2%), the most frequent being PEP, while no procedure-related mortality occurred. The recurrence rate (5.4%) was comparatively low, reflecting effective initial clearance and diligent follow-up. Multiple stones and prolonged hospital stay were identified as significant predictors of complications, underscoring the need for individualized patient assessment and meticulous procedural planning. This study adds valuable local data to regional literature, demonstrating that ERCP can achieve favorable outcomes even in resource-limited settings. Routine use of prophylactic strategies, such as rectal NSAIDs or pancreatic stenting in high-risk patients, along with comprehensive pre-procedural imaging and awareness of anatomical variations, can further minimize complications. Overall, ERCP remains the cornerstone of choledocholithiasis management, offering shorter hospital stays, reduced morbidity, and faster recovery compared to surgical alternatives.
